# The Interrelationship Between Andropause Symptoms, Testosterone Levels, and Lower Urinary Tract Symptoms in Men With Benign Prostatic Hyperplasia (BPH): A Cross-Sectional Study

**DOI:** 10.7759/cureus.88797

**Published:** 2025-07-26

**Authors:** Rishikant Gyani, Apul Goel, Satyanarayan Sankhwar, Bhupendra P Singh, Manoj Kumar, Vivek K Singh

**Affiliations:** 1 Urology, King George's Medical University, Lucknow, IND; 2 Urology, Institute of Medical Sciences, Varanasi, IND

**Keywords:** andropause, benign prostatic hyperplasia (bph), erectile dysfunction, lower urinary tract symptoms (luts), testosterone

## Abstract

Introduction

This study aimed to assess the prevalence of andropause symptoms and testosterone levels in male patients with symptomatic benign prostatic hyperplasia (BPH), as well as to explore the relationship between these factors.

Methods

A total of 183 male patients aged 50 years and older with diagnosed lower urinary tract symptoms (LUTS) due to BPH were included. Andropause symptoms were evaluated using the Male Andropause Symptoms Self-Assessment Questionnaire (MASSQ), while testosterone levels were measured using a chemiluminescent immunoassay. LUTS severity was assessed through the International Prostate Symptom Score (IPSS) and erectile function via the International Index of Erectile Function-15 (IIEF-15). However, the cross-sectional design and it being conducted in a single center limit the study.

Results

The mean andropause symptom score was 57.24 ± 12.62, indicating moderate to severe symptoms. A significant correlation was observed between increasing lower urinary tract symptom (LUTS) severity and higher andropause symptom scores (p < 0.001). Testosterone levels were significantly lower in patients with severe LUTS (285.94 ± 106.9 ng/dL) compared to those with mild LUTS (544.09 ± 109.8 ng/dL). Additionally, comorbidities such as diabetes mellitus and hypertension were associated with increased severity of both LUTS and erectile dysfunction (ED).

Conclusions

The study highlights a significant prevalence of andropause symptoms in men with BPH, emphasizing the correlation between declining testosterone levels and LUTS severity. These findings underscore the importance of comprehensive assessments and management strategies that address both hormonal deficiencies and urinary health in older men.

## Introduction

Benign prostatic hyperplasia (BPH) is a common condition affecting older men, characterized by the noncancerous enlargement of the prostate gland, which leads to a variety of lower urinary tract symptoms (LUTS) [1]. These symptoms can significantly impact a man's quality of life, including urinary frequency, urgency, nocturia, and weak urinary flow. The prevalence of BPH increases with age, affecting approximately 50% of men in their 50s and up to 90% of men over 80 years [2]. As men age, they often experience physiological changes, including hormonal fluctuations, particularly in androgen levels, with the most significant change being the gradual decline in testosterone production, a phenomenon commonly referred to as andropause or late-onset hypogonadism [3]. This decrease in testosterone can manifest in various symptoms, including reduced libido, erectile dysfunction (ED), fatigue, depression, and decreased muscle mass. The relationship between andropause symptoms and BPH is increasingly recognized, yet the interplay between these conditions remains underexplored [4].

The study shows that men with LUTS due to BPH often report a higher prevalence of ED and sexual dysfunction, which are closely linked to the physiological changes associated with aging and declining testosterone levels. The International Index of Erectile Function-15 (IIEF-15) is commonly used to assess erectile function, providing a standardized approach to quantify the severity of ED [5]. Understanding the relationship between BPH, andropause symptoms, and testosterone levels is essential for developing effective management strategies that address both urinary and sexual health. The decline in testosterone not only affects sexual function but also can exacerbate LUTS. Studies have shown that low testosterone levels are associated with the increased severity of LUTS, leading to a vicious cycle where urinary symptoms further impact sexual health and overall well-being [6]. Moreover, comorbidities such as diabetes mellitus and hypertension, prevalent among older men, have been shown to contribute to both ED and BPH symptoms. The multifactorial nature of these conditions necessitates a comprehensive approach to their management [7].

Despite the growing awareness of the interrelationships among BPH, andropause symptoms, and testosterone levels, there remains a paucity of data specifically examining these associations in the North Indian population. This demographic is particularly relevant given the cultural and socioeconomic factors that may influence health-seeking behaviors and attitudes toward sexual health among older men. We had explored the impact of comorbid conditions on the severity of these symptoms, contributing to a more significant understanding of how these factors interact in older men.

## Materials and methods

Study design and participants

This study was conducted at the Department of Urology, King George's Medical University, Lucknow, India, from January 2020 to December 2022. The study was approved by the Institutional Ethics Committee of King George's Medical University (approval number: 1051/Ethics/2020), and written consent was obtained from the patients. A total of 183 male patients aged 50 years and older were enrolled, with inclusion criteria requiring a diagnosis of LUTS attributed to BPH and informed consent to participate. Patients with a history of transurethral resection of the prostate, prostate cancer, current treatment for erectile dysfunction, and significant psychiatric or medical conditions were excluded from the study. By using validated assessment tools such as the Male Andropause Symptoms Self-Assessment Questionnaire (MASSQ) and measuring serum testosterone levels, we are focused on elucidating the relationship between andropause symptoms and LUTS severity.

Inclusion criteria

Male patients aged 50 years and above, diagnosed with LUTS attributable to BPH, and with the ability and willingness to provide informed consent and complete questionnaires were included in the study.

Exclusion criteria

Patients with a history of transurethral resection of the prostate (TURP), the presence of prostate cancer or neurogenic bladder, ongoing treatment for erectile dysfunction, and major psychiatric illness or any other systemic disease likely to confound results (e.g., end-stage renal/liver disease and uncontrolled diabetes) were excluded from the study

Assessment tools

LUTS severity was assessed using the International Prostate Symptom Score (IPSS). Erectile function was evaluated using the IIEF-15. Andropause symptoms were measured using the MASSQ. Each questionnaire was administered in the patients' preferred language by trained personnel.

Laboratory investigations

Total serum testosterone levels were determined using a chemiluminescent immunoassay, with reference ranges per institutional standards. Serum prostate-specific antigen (PSA) levels were measured using an enzyme-linked immunosorbent assay. All samples were collected between 8:00 and 10:00 AM to minimize diurnal variation.

Statistical analysis

Data were analyzed using SPSS version 26.0 (IBM Corp., Armonk, NY). Descriptive statistics were used to summarize demographic and clinical characteristics, with means and standard deviations reported for continuous variables. The normality of data was assessed using the Shapiro-Wilk test. Comparisons between groups were performed using independent sample t-tests for normally distributed data and Mann-Whitney U tests for nonparametric data. Pearson and Spearman correlation coefficients were calculated to assess the relationships between andropause symptoms, testosterone levels, and LUTS severity, with a significance level set at p < 0.05.

## Results

Patient characteristics

A total of 183 male patients aged 50 years and older with benign prostatic hyperplasia (BPH) were included in the study. The mean age of the participants was 63.13 ± 8.98 years. The comorbid conditions included diabetes mellitus, hypertension, and coronary artery disease, as detailed in Table 1.

**Table 1:** Baseline Characteristics of Patients With BPH (n = 183) Values are represented as mean ± standard deviation (SD) for continuous variables and frequency (percentage) for categorical variables. "Other comorbidities" include diabetes and other chronic systemic illnesses LUTS, lower urinary tract symptoms; BPH, benign prostatic hyperplasia

Parameter	Value
Mean Age (Years)	63.13 ± 8.98
Age Distribution	
50-59 years	34.4% (63/183)
60-69 years	38.3% (70/183)
70-79 years	21.3% (39/183)
≥80 years	6.0% (11/183)
Comorbidities	
Other Comorbidities	19.7% (36/183)
Hypertension (HTN)	20.8% (38/183)
Coronary Artery Disease (CAD)	7.7% (14/183)
Severity of LUTS	
Mild LUTS	36.6% (67/183)
Moderate LUTS	33.8% (62/183)
Severe LUTS	29.5% (54/183)

Andropause symptoms in BPH patients

Among the 183 male patients with benign prostatic hyperplasia (BPH), the severity of andropause symptoms was assessed using the Male Andropause Symptoms Self-Assessment Questionnaire (MASSQ). The mean score for andropause symptoms was 57.24 ± 12.62, indicating a moderate to severe prevalence of andropause among the population. As shown in Table 2, patients with more severe lower urinary tract symptoms (LUTS) exhibited significantly higher andropause symptom scores (p < 0.001).

**Table 2:** Andropause Symptom Scores and LUTS Severity in BPH Patients (n = 183) Values are presented as mean ± SD. The severity of LUTS was assessed using the International Prostate Symptom Score (IPSS), and andropause symptoms were measured using the Male Andropause Symptoms Self-Assessment Questionnaire (MASSQ). Statistical comparison was done using ANOVA; p < 0.05 was considered statistically significant LUTS, lower urinary tract symptoms; SD, standard deviation; BPH, benign prostatic hyperplasia

LUTS Severity	Mean Andropause Symptom Score (±SD)
Mild LUTS (n = 67)	51.3 ± 11.7
Moderate LUTS (n = 62)	56.8 ± 12.2
Severe LUTS (n = 54)	63.9 ± 10.6
P-value	<0.001

Testosterone levels and their association with andropause symptoms

Serum testosterone levels were significantly associated with the severity of andropause symptoms. The mean testosterone level across the cohort was 483.65 ± 155.26 ng/dL, with a substantial decline observed in patients with more severe LUTS and andropause symptoms. As shown in Table 3, patients with severe andropause symptoms had markedly lower testosterone levels, particularly in those with severe LUTS (p < 0.001).

**Table 3:** Testosterone Levels and the Severity of LUTS in BPH Patients (n = 183) Data are presented as mean ± SD. Statistical analysis was performed using ANOVA to compare testosterone levels across LUTS severity groups. P < 0.05 was considered statistically significant LUTS, lower urinary tract symptoms; SD, standard deviation; BPH, benign prostatic hyperplasia

LUTS Severity	Mean Testosterone Level (ng/dL) (±SD)
Mild LUTS (n = 67)	544.09 ± 109.8
Moderate LUTS (n = 62)	456.23 ± 166.7
Severe LUTS (n = 54)	285.94 ± 106.9
P-value	<0.001

Erectile dysfunction and comorbidities about andropause symptoms

The study also assessed the severity of erectile dysfunction (ED) and its association with andropause and testosterone levels. A significant portion of patients with severe LUTS experienced moderate to severe ED. As shown in Table 4, 39.9% of patients had moderate ED, while 9.8% had severe ED. Additionally, comorbidities such as diabetes mellitus and hypertension were significantly associated with higher andropause symptom scores (p < 0.05).

**Table 4:** Erectile Dysfunction (ED) and Comorbidities in BPH Patients (n = 183) *Data are presented as mean ± SD for continuous variables and percentage (%) for categorical variables. P-values were calculated using ANOVA for continuous data and chi-square tests for categorical variables. P < 0.05 is considered statistically significant BPH, benign prostatic hyperplasia; SD, standard deviation

Variables	Mild ED (n = 92)	Moderate ED (n = 73)	Severe ED (n = 18)	P-value
Mean Age (Years)	61.2 ± 6.4	63.7 ± 7.8	66.1 ± 9.1	<0.001
Diabetes Mellitus (%)	27.2%	13.7%	5.6%	0.027*
Hypertension (%)	28.3%	12.3%	16.7%	0.039*
Testosterone Level (ng/dL)	544.09 ± 109.8	456.23 ± 166.7	285.94 ± 106.9	<0.001

## Discussion

This study aimed to investigate the prevalence of andropause symptoms and testosterone levels in male patients with symptomatic BPH. Our findings indicate a significant correlation between the increasing severity of LUTS, declining testosterone levels, and the presence of andropause symptoms in the study population. This highlights the complex interplay between hormonal changes and urinary health in older men, emphasizing the need for integrated management strategies. The results revealed that the severity of andropause symptoms, assessed through the MASSQ, was moderate to severe among the participants. Specifically, the mean andropause symptom score of 57.24 ± 12.62 suggests that many men are experiencing considerable distress due to their symptoms. This aligns with previous studies indicating that aging men often report significant andropause-related symptoms, including fatigue, mood changes, and decreased libido [8,9]. Moreover, the association between andropause symptoms and LUTS severity corroborates findings from earlier research that established a relationship between testosterone deficiency and urinary symptoms in older men [6,10].

Our analysis demonstrated that men with severe LUTS had significantly higher andropause symptom scores, suggesting that urinary dysfunction may exacerbate hormonal changes associated with aging. The IPSS indicated that patients with moderate to severe LUTS often experience not only physical symptoms but also psychological distress, which can further compound the effects of andropause. The finding that testosterone levels were significantly lower in patients experiencing severe LUTS supports existing literature linking low testosterone to the increased severity of urinary symptoms [11,12]. This relationship underscores the importance of hormonal evaluation in men presenting with LUTS, as it may provide insight into their overall health status and quality of life [13,14].

Testosterone is crucial for maintaining sexual function, and its deficiency can lead to ED, which is commonly reported in men with BPH. Total testosterone levels below 300 ng/dL were considered indicative of biochemical hypogonadism, while values between 300 and 1000 ng/dL were regarded as within the normal reference range [15,16]. Our study found that the severity of ED was notably higher in participants with declining testosterone levels, emphasizing that the interplay between BPH and hormonal changes significantly impacts men's sexual health. The IIEF-15 scores indicated that nearly 50.3% of the participants had mild ED, while 39.9% exhibited moderate ED. This prevalence is consistent with other studies, which suggest that ED is common among older men with BPH and is exacerbated by the decline in testosterone levels [5,17].

In addition to hormonal influences, the study identified comorbid conditions, such as diabetes mellitus and hypertension, as significant contributors to the severity of both LUTS and erectile dysfunction. These findings are critical, as they highlight the need for healthcare providers to consider the broader health context of their patients. Patients with diabetes are known to have a higher prevalence of sexual dysfunction and urinary symptoms, and managing these comorbidities is essential for improving overall health outcomes [18]. Furthermore, the study found a notable association between hypertension and the severity of LUTS, suggesting that vascular health plays a role in both urinary and sexual function [19-21].

This study provides valuable insights into the prevalence of andropause symptoms and testosterone levels in men with symptomatic BPH. Our findings underscore the importance of recognizing the interconnectedness of hormonal health, urinary symptoms, and comorbid conditions in older men. An integrated approach that addresses both hormonal deficiencies and urinary dysfunction is essential for enhancing the quality of life for this population. Clinicians should prioritize comprehensive assessments that consider hormonal status when managing patients with BPH and LUTS. Ultimately, further research is needed to explore effective interventions aimed at alleviating andropause symptoms and improving overall health outcomes for men experiencing BPH.

Limitations

This study has several limitations. Its cross-sectional design prevents establishing causal relationships between andropause symptoms, testosterone levels, and LUTS. Conducted at a single tertiary care center, the findings may not be generalized to broader populations. The reliance on self-reported questionnaires introduces potential bias, despite using validated instruments. Additionally, the study did not comprehensively evaluate confounding factors such as lifestyle and psychological well-being. Finally, while the sample size was adequate for preliminary findings, larger multicenter studies are needed to confirm these results and explore the interactions between these variables more thoroughly.

## Conclusions

This study demonstrates the significant prevalence of andropause symptoms in male patients with symptomatic BPH, highlighting a strong correlation with declining testosterone levels. Severe lower urinary tract symptoms were associated with increased andropause symptoms and erectile dysfunction, further exacerbated by comorbidities such as diabetes and hypertension. These findings emphasize the need for comprehensive management strategies that address both hormonal deficiencies and urinary health in older men.
